# An evolutionary signal to fungal succession during plant litter decay

**DOI:** 10.1093/femsec/fiz145

**Published:** 2019-09-07

**Authors:** Sasha Vivelo, Jennifer M Bhatnagar

**Affiliations:** Dept. of Biology, Boston University, Boston, MA 02215, USA

**Keywords:** decomposer fungi, succession, plant litter, fungal communities, evolution, phylogenetic signal

## Abstract

Ecologists have frequently observed a pattern of fungal succession during litter decomposition, wherein different fungal taxa dominate different stages of decay in individual ecosystems. However, it is unclear which biological features of fungi give rise to this pattern. We tested a longstanding hypothesis that fungal succession depends on the evolutionary history of species, such that different fungal phyla prefer different decay stages. To test this hypothesis, we performed a meta-analysis across studies in 22 different ecosystem types to synthesize fungal decomposer abundances at early, middle and late stages of plant litter decay. Fungal phyla varied in relative abundance throughout decay, with fungi in the Ascomycota reaching highest relative abundance during early stages of decay (*P* < 0.001) and fungi in the Zygomycota reaching highest relative abundance during late stages of decay (*P* < 0.001). The best multiple regression model to explain variation in abundance of these fungal phyla during decay included decay stage, as well as plant litter type and climate factors. Most variation in decay-stage preference of fungal taxa was observed at basal taxonomic levels (phylum and class) rather than finer taxonomic levels (e.g. genus). For many finer-scale taxonomic groups and functional groups of fungi, plant litter type and climate factors were better correlates with relative abundance than decay stage per se, suggesting that the patchiness of fungal community composition in space is related to both resource and climate niches of different fungal taxa. Our study indicates that decomposer fungal succession is partially rooted in fungal decomposers’ deep evolutionary history, traceable to the divergence among phyla.

## Teaser

The evolutionary history of fungal decomposers contributes to the structure of complex microbial decomposer communities.

## INTRODUCTION

**Figure 1.**
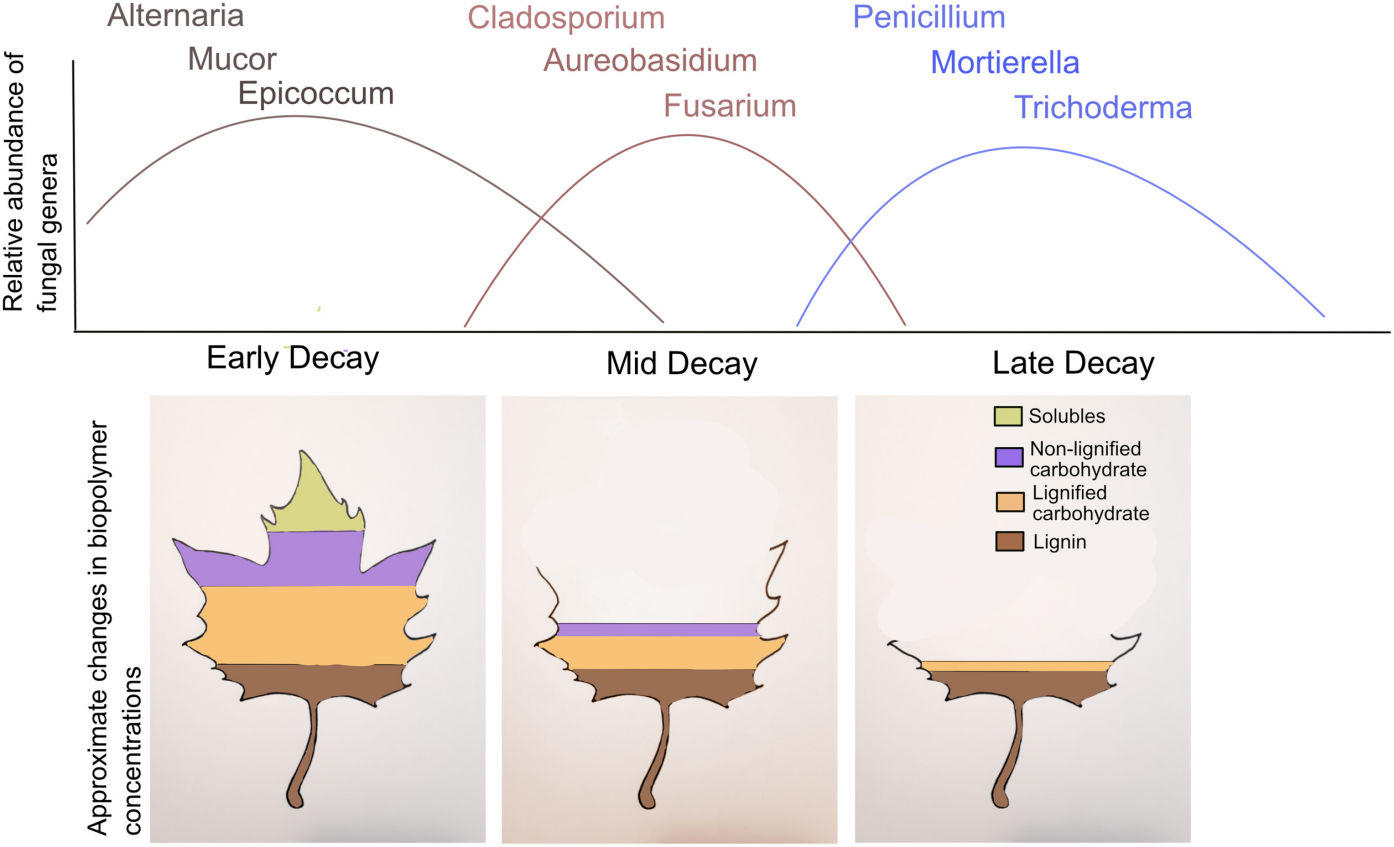
Hypothesized changes in fungal decomposer community composition during litter decomposition (Kjøller and Struwe 2002; Berg and McClaugherty 2014). The concentration of plant biopolymers changes as labile litter chemicals (i.e. soluble carbohydrates) degrade faster than recalcitrant material (i.e. lignin). Lines on the relative abundance graph represent the change in relative abundance of the corresponding color-coded genera, as reported by Kjøller and Struwe (2002).

Decomposer fungi cycle up to 36 Gt carbon (C) per year through soils (Talbot 2017), releasing ∼3× more CO_2_ to the atmosphere annually than human emissions (Lal and Follett 2009; Giardina *et al*. 2014). Nevertheless, we still have a limited understanding of how fungal species in these diverse communities coordinate their activity to drive the decay of dead organic matter (i.e. litter) and influence ecosystem C cycling (Kjøller and Struwe 2002). Fungal decomposer communities show patterns of assembly similar to those observed among plant and animal communities (Rayner and Boddy 1988; Lindner *et al*. 2011; Ottosson *et al*. 2014), where different species become dominant at different points in time (i.e. succession) (Odum 1969). It has been observed anecdotally that closely related fungal species can dominate the same stages of decomposition in different habitats (Fig. 1). If such a phylogenetic pattern of fungal succession exists, it would suggest that the assembly of decomposer communities—and perhaps even the rate and process of decay—could be predicted simply from where fungal species in a decomposer community sit on the evolutionary tree of life. It would also suggest that the biology of decomposer organisms is an important control over their activity in nature, perhaps even more so than environmental factors (Zhang *et al*. 2008; Todd-Brown *et al*. 2012). Fungal succession is one of the most widespread and universal patterns of community assembly and activity in ecosystem ecology (Kjøller and Struwe 2002), so understanding key taxonomic features of this process could bring insight into the biology behind one of the largest fluxes of C and energy through the biosphere.

There are several competing hypotheses about how fungal succession is structured phylogenetically across ecosystems. One longstanding hypothesis is that fungal decomposer succession follows a predictable pattern according to species’ deep evolutionary history, where species in different fungal phyla dominate early and late stages of decay (Frankland 1998; Kjøller and Struwe 2002; Treseder *et al*. 2014). Specifically, fungi in the phylum Ascomycota are thought to proliferate earlier in decay than fungi in the phylum Basidiomycota, because they possess differential capacity for the breakdown of plant biopolymers that change in relative abundance throughout decay (Osono 2007). For example, the Basidiomycetes contain most of the white rot fungi, which are thought to be the primary decomposers of recalcitrant plant biopolymers like lignin (Rayner and Boddy 1988; Morgenstern, Klopman and Hibbett 2008) that have higher concentrations later in decomposition (Berg and McClaugherty 2014). In addition, fungal phyla differ in their inherent growth rates and reproductive strategies; most Ascomycota can reproduce either sexually or asexually and proliferate quickly under favorable circumstances, whereas many Basidiomycota are thought to be obligate sexual reproducers and so may grow more slowly during the decomposition process (Webster and Weber 2007). Such relationships between preferred decay stage and the inherent growth rates of fungi would be consistent with island biogeography theory developed for plants and animals, which suggests that species with high reproduction and growth rates are more likely to establish during the early stages of succession (MacArthur and Wilson 1967; Andrews *et al*. 1987). Nevertheless, it is unclear whether or not the preferred decay stage of fungi (i.e. the stage at which each taxon reaches its highest relative abundance (Mönkkönen *et al*. 2011; Treseder and Lennon 2015; Norros and Halme 2017)) can be traced back to the divergence of fungal phyla. Early studies documented high abundances of Ascomycota during early stages of decay at individual study sites, based on fruiting bodies and culturing of fungal isolates from wood and decomposing litter (Frankland 1966; Aneja *et al*. 2006; Osono 2007; Voříšková and Baldrian 2013). However, other fungal phyla (e.g. lineages in the phyla Zoopagomycota and Mucoromycota) are important decomposers of plant matter (White *et al*. 2006; Mundra *et al*. 2016) and no clear hypothesis has been laid out regarding the decay-stage preference for these lineages.

An alternative hypothesis is that there may be no deep evolutionary roots to fungal succession and instead, fungi may prefer different decay stages based on decay traits that evolve rapidly within lineages. Recent studies of fungi have revealed that functional traits (such as specialization in cellulose degradation) can be overdispersed (i.e. not clustered among closely related taxa) across fungal decomposer lineages (Hibbett and Donoghue 2001; McGuire *et al*. 2010; Treseder *et al*. 2014) and functional traits of fungi are often assigned at the genus level, rather than at higher taxonomic levels (Treseder and Lennon 2015; Nguyen *et al*. 2016). Indeed, important decomposer traits such as white rot or brown rot decay biochemistries can vary among fungal genera within families (Hibbett, Donoghue and Olmstead 2001) and seminal review articles on fungal succession regard fungi as ‘important’ players in early or late stages of litter decomposition in different ecosystems based on their genus-level classifications (Kjøller and Struwe 2002; Osono 2007). For example, one emerging idea is that fungal genera that act as foliar endophytes are some of the earliest-acting decomposer fungi, as they are found in the phyllosphere of live plants and may switch to decomposers after leaf senescence (Promputtha *et al*. 2007; Voříšková and Baldrian 2013). The hypothesis that decay-stage preference of decomposer fungi occurs primarily at the genus level is also consistent with recent studies of plant and animal succession, which suggest that phylogenetic relatedness is often not a sufficient stand-in for similarity of ecological function (Losos 2008; Swenson and Enquist 2009; Purschke *et al*. 2013).

Physical and/or environmental factors may also interact with fungal biology to shape community succession, such that the taxonomic patterns of succession could be largely unique to individual habitats, rather than universal across systems. While certain fungal genera have been described as major players in decomposition (e.g. *Aureobasidium*, *Cladosporium*, etc. (Kjøller and Struwe 2002; Osono 2007)), the data that support this claim come from mixed hardwood/coniferous forest ecosystems that have similar climate conditions and plant species. Other biomes can be dominated by different genera of decomposer fungi (e.g. *Stigmina* in Mediterranean ecosystems (Pasqualetti *et al*. 1999), *Nigrospora* and *Pestalotiopsis* in tropical ecosystems (Osono *et al*. 2009), and *Phoma* in temperate grassland ecosystems (Mincheva *et al*. 2014)), perhaps because fungal species have different climate tolerances (Hawkes *et al*. 2011; Crowther and Bradford 2013; Looby and Treseder 2018). Furthermore, plant species can impact microbial decomposer community composition (Bollen 1953; Melillo, Aber and Muratore 1982; Kattge *et al*. 2011; Urbanová, Šnajdr and Baldrian 2015; Bruder *et al*. 2016; Sayer *et al*. 2017) as can plant tissue type (e.g. leaf vs. stem) (Fogel and Cromack 1977), potentially because plant species and tissues vary in the initial chemical composition of their litter (Bollen 1953; Fogel and Cromack 1977; Cornwell *et al*. 2008; Meier and Bowman 2010; Kattge *et al*. 2011) and fungal species often vary in their preference for plant biopolymer resources (McGuire *et al*. 2010; Sinsabaugh *et al*. 2014). Indeed, recent observations indicate that soil fungi can exhibit strong endemism at the species level (i.e. ∼85% of fungal species turnover in soil samples that are 1000 km apart), as well as at the genus and family levels (Talbot *et al*. 2014). If taxonomic patterns of fungal succession are entirely unique to individual biomes, it would suggest that our most widely used predictive models of terrestrial C cycling, which ignore biological information about decomposer organisms and instead use climate and plant characteristics to parameterize litter decay rates (Manzoni and Porporato 2009), are sufficient to represent fungal activity in the terrestrial C cycle. On the other hand, if a common taxonomic pattern of fungal succession is detectable across regions with different climate and resource environments when we control for climate and resource factors, it suggests that terrestrial C cycle models may benefit from explicitly representing fungal taxonomy, as changes in fungal community composition could disrupt the decay process.

In this study, we aimed to answer two overarching questions about the taxonomic patterns and potential drivers of fungal decomposer succession across ecosystems: (i) Is there a common taxonomic pattern of fungal succession across ecosystems (i.e. are the same fungi dominant at the same stages of decay regardless of habitat)? and (ii) If so, at what point in evolutionary history does a taxonomic pattern of fungal succession emerge? To answer these questions, we conducted a meta-analysis of published fungal decomposer succession studies, including studies that have used recently developed high-throughput DNA sequencing technologies, as well as studies that used older culturing and microscopy techniques. Due to longstanding precedent in the literature, we hypothesized that there is a common taxonomic pattern to fungal decomposer succession across different ecosystems and that the pattern exists at the phylum level, where different fungal phyla prefer (i.e. reach their highest relative abundance at) different stages of decay. We also hypothesized that there would be a taxonomic pattern to succession across studies that emerged at the genus level, due to different resource specializations among fungal genera (Hanson *et al*. 2008). However, we hypothesized that, because fine taxonomic groups of fungi exhibit endemism (i.e. species and genera) (Talbot *et al*. 2014), a taxonomic pattern to succession across ecosystems would be detectable more so at coarser taxonomic levels (i.e. phyla) than at finer levels of taxonomy (i.e. genera).

## METHODS

### Study dataset

We conducted a meta-analysis of fungal succession during plant litter decomposition using published studies of fungal taxon abundances reported for particular time points in the decay process. Candidate studies were identified by searching literature from 1950–2018 using the terms ‘fungal succession leaf litter’, ‘fungal succession decay’, ‘fungal succession waste’, ‘mycological succession’, ‘fungi succession’ and ‘fungal diversity’ in Web of Science (ISI). We then screened the methods and results section to select primary research articles that included (i) relative abundance of individual fungal taxa (at the species level) during experimental decomposition of plant litter, (ii) a taxonomic ID (i.e. genus, species name) and relative abundance measure of individual fungi matched to a specific time point since onset of decay (i.e. after time 0) and (iii) % litter mass loss at each specific time point during decay (i.e. exact time of the onset of decay was known, and litter mass was measured at onset of decay and at each sampling time point). Percent litter mass loss follows a well-studied trajectory and is regarded as a proxy for decay stage (Berg and McClaugherty 2014). A total of 1700 candidate fungal succession studies were considered and 22 studies met the criteria for inclusion (Table S1, Supporting Information). Studies spanned four biomes (temperate, tropical or subtropical, Mediterranean and boreal) and four broad plant categories: deciduous trees (11 studies), coniferous trees (4 studies), non-coniferous evergreens (2 studies), grasses and herbs (5 studies) and shrubs (2 studies), as well as four plant tissue types: leaf litter only (18 studies), stem and leaf (2 studies), leaf and root (1 study) and reproductive tissue (beech cupules; 1 study). One of three methods was used to quantify fungal taxon abundances: culturing fungi from decomposed litter (17 studies), sequencing fungal rDNA extracted from litter (4 studies used ITS rDNA for identification and 1 study used 18S rDNA) and counting the incidence of a taxon on decomposing litter samples using microscopy (1 study). For culture studies, taxonomy was assigned to unique colonies or conidia. Relative abundance of each taxon was determined by calculating the percentage of litter samples that yielded each species (11 studies), counting the number of conidia per unit dry litter mass (6 studies) or estimating the population using the plate count method (1 study). Sequencing studies measured taxon abundances as raw counts of an operational taxonomic unit (OTU), which were constructed in each study by using a 97% sequence similarity cutoff and rarefaction (Table S1, Supporting Information). We considered OTUs to be individual fungal taxa, because the 97% sequence similarity cutoff is known to approximately delineate fungal species (Smith *et al*. 2007). Studies reported relative abundance data from 5 to 792 fungal species (culture/microscopy studies: average 24, SD 17; sequence studies: average 414, SD 356). We assigned taxonomy to individual species using the original taxonomic classifications from each study and we assigned functional guild (white rot/brown rot, endophyte/non-endophyte and yeast/filamentous) to species according to published functional guild and life-strategy observations (Tedersoo, May and Smith 2010; Kurtzman, Fell and Boekhout 2011). While the accepted taxonomy for some groups has changed (i.e. Zygomycota is now know to comprise the phyla Zoopagomycota and Mucoromycota (Spatafora *et al*. 2016)), we use the older term ‘Zygomycota’ to maintain the taxonomic assignments made in early culture and microscopy-based studies and to keep consistent taxonomic names across studies, as most culture studies included in our meta-analysis were performed before the taxonomic re-assignment of this phylum in 2016. The taxonomic distribution in the final dataset included 7 phyla, 27 classes, 125 orders, 277 families and 835 genera of fungi, with 1 to 37 species per genus (average 2, SD 3).

### Data acquisition

Fungal taxonomic identification, the relative abundance of each taxon at each sampling time point and the % litter mass loss at each sampling time point were obtained directly from each study manuscript. In cases where fungal abundance and % litter mass loss were provided graphically, data were extracted using WebPlotDigitizer (Rohatgi 2014). Initial litter % C and N data for plant species were either obtained directly from the study manuscript (in the case of three of the four sequence studies) or downloaded from the TRY database (Wilson, Baldocchi and Hanson 2000; Kattge *et al*. 2011; Yguel *et al*. 2011; Wright and Sutton-Grier 2012), which provides data aggregated from published studies for a plant type. Initial C was available for 13 plant species (across eight studies) and initial N was available for nine plant species (across five studies). Mean annual temperature and mean annual precipitation were obtained for each study location, based on latitude and longitude of each study site, from WorldClim's bioclimatic variables (Fick and Hijmans 2017) using R's raster package (Hijmans 2016).

### Data normalization and aggregation

Data were normalized in each study to the % relative abundance of the total fungi detected in each sample ([detected abundance of each taxon in each sample]/[total abundance of all taxa detected in the sample] × 100). We chose to normalize data from each study in this way because relative abundance is an informative measure of community composition in both classic ecological studies (Peet 1974; James and Rathbun 1981) and recent microbial sequence studies (Koenig *et al*. 2011; Fierer *et al*. 2012; Koren *et al*. 2013). Therefore, we normalized data from all studies, regardless of measurement methodology (culture, microscopy or DNA sequence) using this procedure. Data points for use in all downstream statistical analyses were calculated by averaging data by study, sampling time point, litter plant species and litter tissue type (i.e. taxon abundances are averaged over a sampling time point for a tissue type within a plant species within a study). This data aggregation resulted in a total of 1 to 23 data points per study (average 7, SD 5) (culture/microscopy studies: range 1 to 23, average 7, SD 5; sequence studies: range 6 to 15, average 9, SD 4), 7 to 162 data points per fungal phylum (average 54, SD 53 data points per study) and 1 to 15 data points per fungal genus (average 9, SD 5 data points per study). Because using several observations from each study violated the assumption of independence among data points that was necessary for our subsequent statistical analyses, we accounted for the effect of study by weighting data points by inverse variance following Vevea and Hedges’ method (Vevea and Hedges 1995; Marín-Martínez and Sánchez-Meca 2010) prior to phylogenetic and statistical analyses.

### Phylogenetic tree construction

To test for a phylogenetic signal to fungal succession, we built a phylogenetic tree representing taxa in all fungal genera detected across the 22 studies using 28S sequences downloaded from the SILVA ribosomal RNA database (version 128) (Quast *et al*. 2012). Sequences were downloaded for 1–3 individual species in each of 538 genera, and a single high-quality sequence was selected as a representative of the genus. Selected sequences were aligned to the SILVA SEED LSU nucleotide alignment and used to construct a phylogenetic tree in RAxML using the GTRGAMMA model of nucleotide substitution rates, with 100 bootstrap replicates followed by ML optimization (Stamatakis 2014). The final phylogeny was visually checked against the currently accepted fungal phylogeny according to James *et al*. (2006), and Blackwell *et al*.’s *Fungal Tree of Life Web Project* (James *et al*. 2006; Blackwell *et al*. 2012).

### Statistics

We tested the hypothesis that a common taxonomic pattern to fungal decomposer succession exists across ecosystems by analyzing relationships between the relative abundance of fungal taxa, decay stage (represented by % original litter mass loss) and other environmental variables (e.g. climate, plant category) across studies. This analysis was conducted in two ways: (i) by testing for relationships using the relative abundance of fungi identified to the phylum level and the genus level without accounting for phylogenetic relatedness (Fernandez *et al*. 2017) and then (ii) testing for a phylogenetic signal to preferred decay stage of each fungal genus (Mönkkönen *et al*. 2011; Treseder *et al*. 2014; Norros and Halme 2017). For the first (non-phylogenetic) analysis, we regressed relative abundance of fungal taxa against % mass loss using a random effects generalized linear multiple regression model (Yee and Mitchell 1991) that is commonly used to model relative abundance data (Warton *et al*. 2015). Separate regression models were run for either relative abundance of individual fungal phyla or relative abundance of individual fungal genera as the dependent variable. In addition to % mass loss, other environmental variables (mean annual temperature, mean annual precipitation, litter plant category and litter tissue type) were used as independent covariates in each model. To test the hypothesis that initial litter chemistry was responsible for correlations between plant category or litter tissue type and fungal taxon relative abundances, we replaced plant category and tissue type with initial litter C and N in any multiple regression models for which plant category or tissue type showed a significant relationship with fungal taxon relative abundance. To obtain the final multiple regression model that best explained variance in taxon relative abundances, a combination of forward and backward selection (Yan and Su 2009) was applied using the stepAIC function in R's MASS package (Venables and Ripley 2002). We focused our phylum-level analysis on three major fungal phyla (Ascomycota, Basidiomycota and Zygomycota), as they are the most frequently observed, the most abundant and believed to be the most critical in the decay process (Kjøller and Struwe 2002). To investigate the effect of measurement method (sequence vs. culture) on fungal relative abundances, we analyzed the sequence and culture data for each phylum both separately and together. While Ascomycota were detected frequently in both study types, Basidiomycota and Zygomycota were so infrequently detected in culture studies that culture study data provided insufficient statistical power to be informative for these two phyla. Therefore, data from sequence studies only was used to determine the final models for Basidiomycota and Zygomycota. However, for the Ascomycota, analyses of DNA sequence data alone, culture data alone and sequence and culture data together resulted in the same final model (Table S2, Supporting Information). Therefore, in order to use as much information as possible in our hypothesis testing, we analyzed data from all methodologies together for the Ascomycota. We focused our genus-level analysis on the 34 fungal genera detected in four or more biomes, as well as 5 additional fungal genera with succession patterns frequently reported in individual studies (39 total). We regressed relative abundance of each genus against % mass loss and environmental variables (i.e. mean annual temperature, mean annual precipitation, litter tissue type and litter plant category) using a generalized additive model (GAM) (Wood 2007) to allow for testing of non-symmetric response curves (Yee and Mitchell 1991). Because some genera were identified in very few studies, we used both sequence and culture data to maximize statistical power in genus-level regressions. When performing regressions for these 39 common and/or well-studied genera, we chose not to adjust *P*-values for multiple comparisons because adjustments increase the risk of false-negative errors and could result in a failure to flag important correlations for future study (Rothman 1990; Gelman, Hill and Yajima 2012; Crowther *et al*. 2015); this is especially true in the case of the analysis of nonrandom natural/ecological observations (Rothman 1990; Crowther *et al*. 2015).

For the second (phylogenetic) analysis, we tested for a phylogenetic signal to preferred decay stage in two ways. First, we determined the % mass loss of each genus’ highest relative abundance, then fit a likelihood model for evolution of preferred decay stage to the phylogenetic tree of fungal genera using the fitDiscrete function in R's geiger package (Harmon *et al*. 2008) and compared it to a null model using a chi squared test. Second, we determined the taxonomic level at which most variation in preferred decay stage can be detected using contribution index calculated with the Phylocom software (Webb, Ackerly and Kembel 2008). In this analysis, we also used the % mass loss of each genus’ highest relative abundance as the dependent variable, to reflect the ‘preferred decay stage’ of a genus. The contribution index describes each node's proportion of contribution to the total variation of a given trait across a phylogenetic tree. To test for whether the contribution index of each taxonomic level (e.g. class, order and genus) to variation in preferred decay stage among fungi differed significantly from other taxonomic levels, we ran an ANOVA with taxonomic level as the independent variable and contribution index as the dependent variable. Significant differences among taxonomic levels in contribution index were detected using a Tukey post-hoc test (Enderlein 1970; R Development Core Team 2011).

## RESULTS

**Figure 2.**
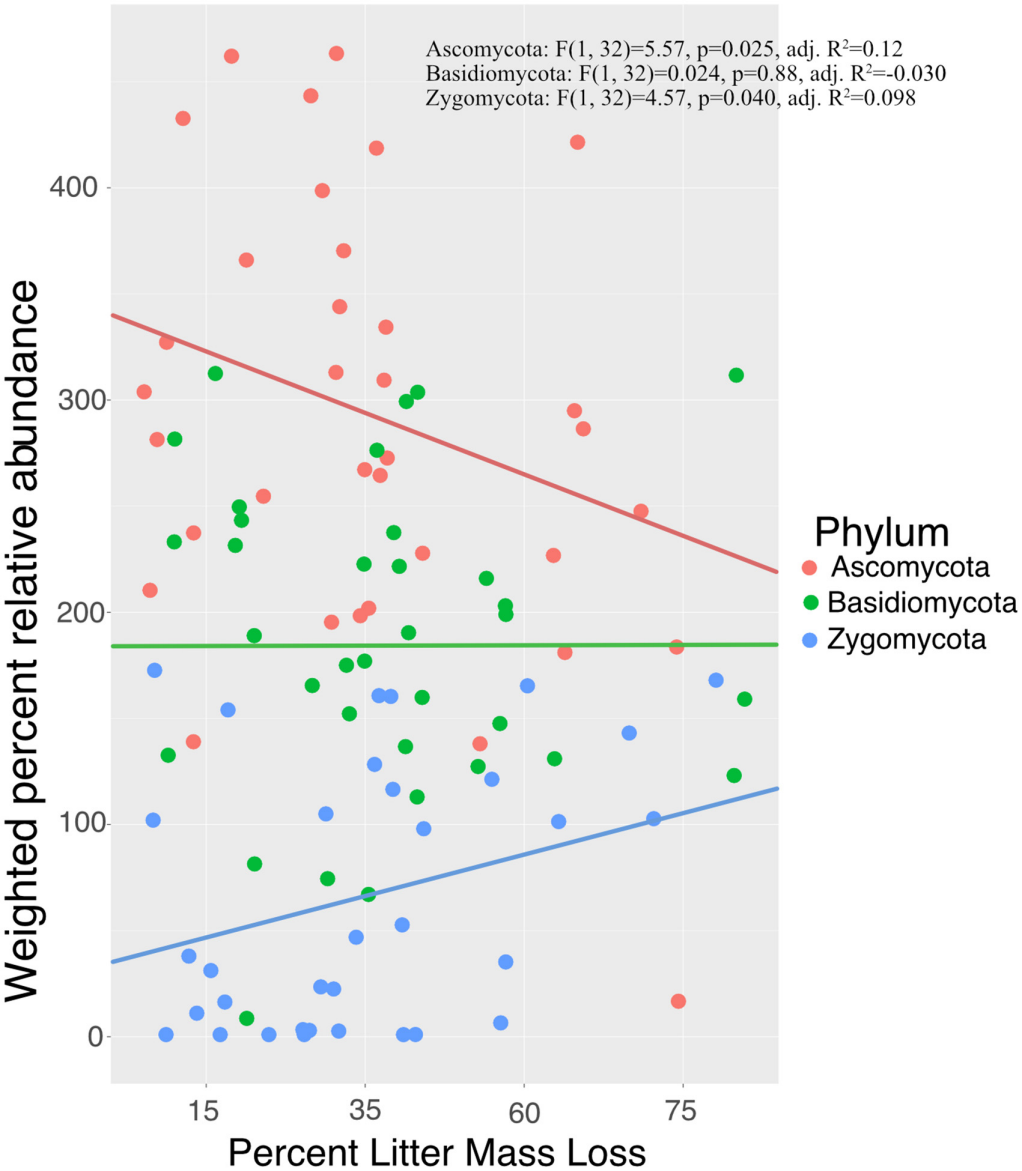
Relationship between relative abundance of major decomposer fungal phyla and % litter mass loss during decay. Each point represents individual sampling time point on a litter type in a published sequence study (*n* = 34 per phylum). Lines represent single linear regression model trend lines. *P*-values and adjusted *R*^2^ values represent the results of the single regression model for each phylum against % litter mass loss.

**Figure 3.**
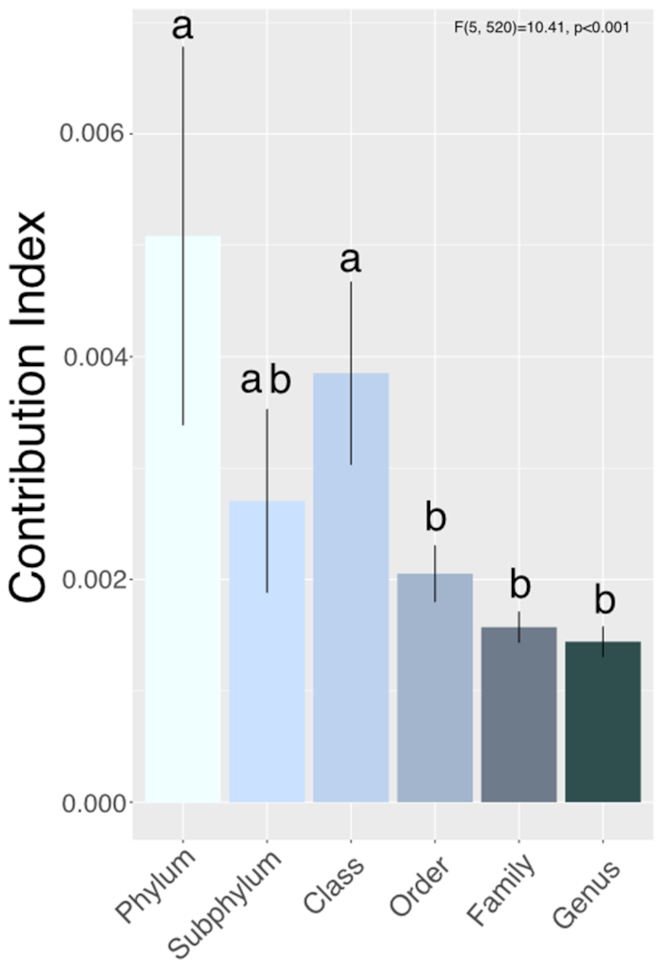
The contribution of each taxonomic level to the total variation in preferred decay stage across the phylogenetic tree. Bars represent the mean contribution index, calculated using aotf in Phylocom (Webb, Ackerly and Kembel 2008), for each taxonomic level (*n* = 9–207). Higher bars represent greater variation in preferred decay stage at that taxonomic level. Error bars represent standard errors around the mean.

We found a common taxonomic pattern of succession across studies at the phylum level, where the relative abundance of Ascomycetes correlated negatively with % litter mass loss and Zygomycetes correlated positively with % mass loss (Fig. 2 ). However, the relative abundance of Basidiomycete fungi did not vary significantly throughout decay. We also found a significant phylogenetic signal to preferred decay stage of fungi (*P*< 0.001) (Figure S1, Supporting Information). Partitioning the variance in relative abundance of fungi across different taxonomic levels in the phylogenetic tree (using the CI metric), we found that the greatest variation in preferred decay stage occurred at the phylum and class levels (*P*< 0.001) (Fig. 3). Fungi in the Ascomycota were consistently of highest relative abundance across studies, compared to fungi in other phyla (Figure S2, Supporting Information), and correlated negatively with mean annual precipitation and temperature (Figure S3, Supporting Information). Relative abundance of Ascomycetes also varied by plant category and litter tissue type, having the highest relative abundance on coniferous plants, leaves and stems (Figure S4, Supporting Information). Zygomycete relative abundance correlated positively with mean annual precipitation, as well as with plant category and litter tissue type (Figures S3 and S4 and Table S2, Supporting Information), such that the relative abundance of fungi in the Zygomycota was highest on litter from deciduous plants and grasses/herbs. For both Ascomycota and Zygomycota, relative abundance correlated with initial litter C and N (Figure S4 and Table S3, Supporting Information), such that relative abundance of Ascomycota correlated positively and Zygomycota correlated negatively with initial C and N of litters. Relative abundance of Basidiomycetes did not correlate with any factor used in our analysis (Table S2, Supporting Information). Insufficient data was available to determine relationships between abundance of other phyla (Chytridiomycota, Rozellomycota and Neocallimastigomycota) and any predictors (% litter mass loss, litter plant and tissue type, temperature and precipitation) across studies.

**Figure 4.**
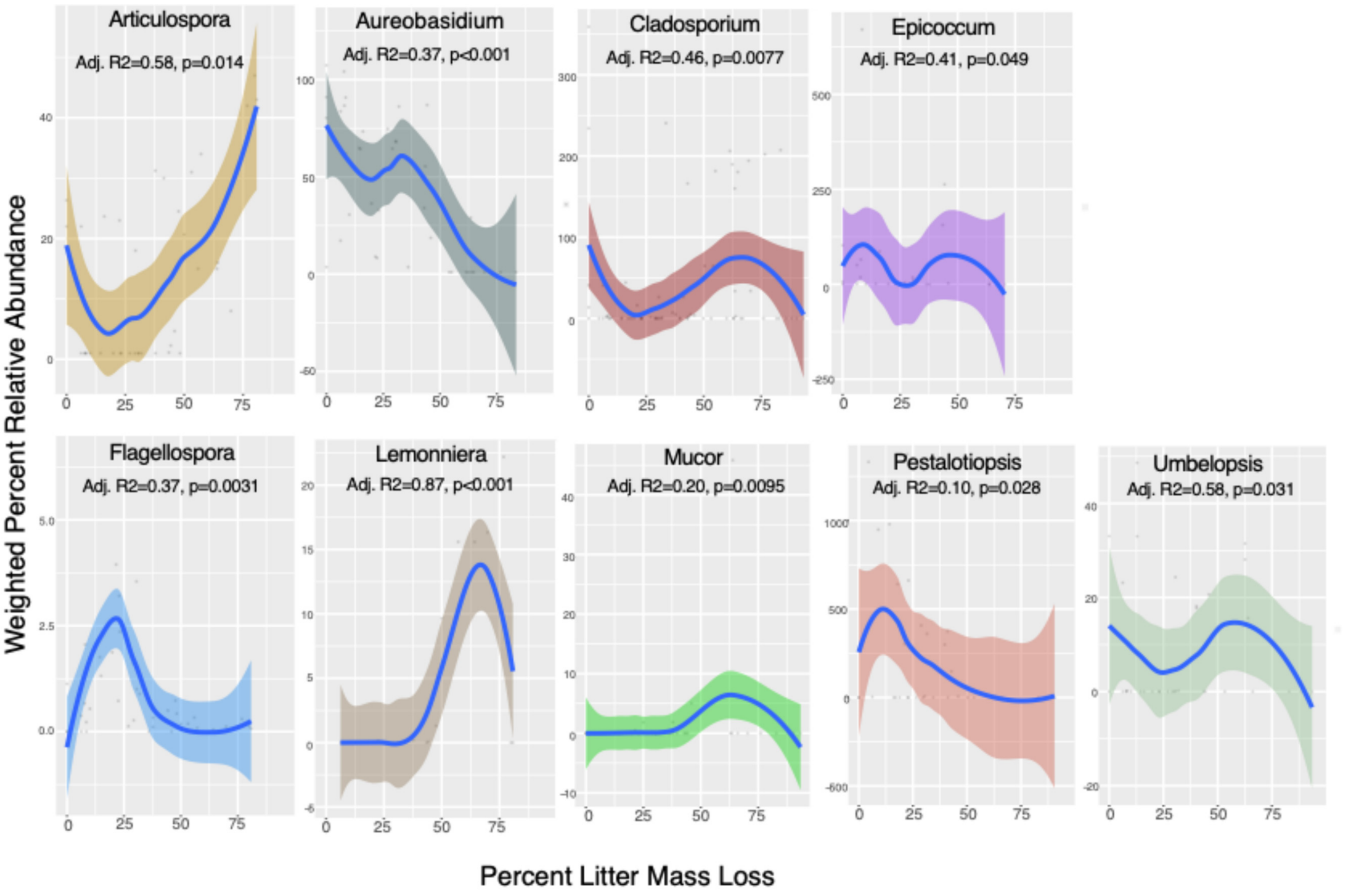
Smooth curves illustrating significant relationships between weighted relative abundance of genera and % litter mass. *P*-values and adjusted *R*^2^ values represent the results of generalized additive multiple regression models. Shaded areas represent 95% confidence interval around the mean.

The relative abundance of 9 of the 34 most cosmopolitan genera (identified in 4+ biomes) varied significantly by % litter mass loss (Fig. 4). Five genera had highest relative abundance during early decay (*Pestalotiopsis*, *Epicoccum*, *Umbelopsis*, *Aureobasidium and Flagellospora)*, while *Cladosporium* reached two separate peaks in relative abundance: one in early decay and the other in late decay. The other three genera had highest relative abundance during late decay (*Mucor, Articulospora* and *Lemonniera*). In addition, environmental factors correlated significantly with the relative abundance of the most cosmopolitan genera; plant category correlated significantly with the relative abundance of *Trichoderma, Cladosporium, Mucor, Penicillium, Umbelopsis, Cryptococcus, Geniculosporium, Gyoerffyella* and *Lachnum*, and tissue type correlated significantly with the relative abundance of *Phoma, Xylaria, Cryptococcus, Epicoccum, Lachnum* and *Tetracladium*. While more genera were detected in DNA sequence studies than in culture-based studies (480 in culture studies vs. 677 in sequence studies), about two-thirds as many unique genera were found in the culture studies as were found in the sequence studies. Taking both sequence and culture studies into account (836 genera in total), decay-stage preference of fungal genera did not vary by functional guild (e.g. white rot, brown rot, foliar endophytes) (Figures S5 and S6, Supporting Information). However, the white rot members of Basidiomycota varied significantly by plant category and were particularly high on coniferous plant litter (Figure S5, Supporting Information).

## DISCUSSION

Fungal decomposer community composition has frequently been observed to change through decay time (Frankland 1966; Kjøller and Struwe 2002; Voříšková and Baldrian 2013; Treseder *et al*. 2014), yet it is not clear which biological or environmental factors drive fungal decomposer succession or how they vary across ecosystems. We hypothesized that there would be a common taxonomic pattern of fungal succession across ecosystems, and that this pattern would be rooted in the evolutionary history of fungi, where different fungal phyla prefer early vs. late stages of decay across ecosystems. In support of this hypothesis, we found that fungi in the phylum Ascomycota preferred early decay stages, while fungi in the phylum Zygomycota preferred later decay stages (Fig. 2), suggesting that a set of fundamental biological principles underlie fungal activity in nature. However, we also found that the relative abundance of fungi in the Basidiomycota remained relatively constant throughout decay. Moreover, we found that the relative abundance of many fungal lineages, including most fungal genera, do not vary predictably with % mass loss during decay when taking climate and plant litter factors into account. These observations support the idea that different fungal taxa often have unique resource and climate niches, a feature of the kingdom which may contribute to the regional endemism of terrestrial fungal communities (Talbot *et al*. 2014). Nevertheless, a handful of widely distributed fungal genera do have significant decay-stage preferences (Fig. 4) and may have important roles in shaping fungal community composition or the decomposition process at each stage of decay.

Several aspects of fungal biology may contribute to decay-stage preferences of certain fungal phyla during litter decomposition. If Ascomycetes primarily target labile C sources during decay, it may account for their high relative abundance early in decomposition and allow them to persist at high relative abundance throughout the decay process. Furthermore, asexually-reproducing species in the Ascomycota could have faster rates of growth and establishment relative to species in the Basidiomycota due to the lower energy cost of asexual reproduction (Heitman, Sun and James 2013), as similar connections between growth rate and dominance has been found for plant and animal communities (MacArthur and Wilson 1967; Odum 1969; Bazzaz 1979). While the Basidiomycota include specialized, efficient lignin degraders (Morgenstern, Klopman and Hibbett 2008; Floudas *et al*. 2012), we found that lignin-degrading fungal genera (i.e. white rot fungi) did not vary in relative abundance during decay (Figure S5, Supporting Information). Recent studies indicate that lignin degradation occurs throughout decay (Klotzbücher *et al*. 2011; He *et al*. 2016), which may allow lignin-degrading Basidiomycetes to support consistent growth from early through late decay.

We found clear bias in reporting the relative abundance of fungal lineages across studies based on methodology, which may have impacted the strength of decay-stage preference we observed for different fungal lineages. For example, the relative abundance of Basidiomycota and Zygomycota were higher in DNA sequence studies than culture studies, possibly because fungi in the Basidiomycota and Zygomycota are difficult to culture than fungi in the Ascomycota (Tanabe *et al*. 2000; Hunt *et al*. 2004; Rizzo and Pang 2005; Kjøller and Rosendahl 2014). The relatively low number of published high-throughput DNA sequence studies, and the fact that sequence studies may detect DNA from inactive species (Carini *et al*. 2017), may have led to the significant but weak correlation between relative abundance of phyla with % litter mass loss that we observed (Fig. 2). Nevertheless, we found that methodology had no impact on trends for lineages that are easily cultured (i.e. the Ascomycota), indicating that changes in relative abundance detected via DNA sequencing may reflect true shifts in the decomposer community throughout decay.

The relative abundance of fungal phyla during decay correlated with other environmental factors besides % mass loss (Figures S3 and S4 and Table S2, Supporting Information), supporting the idea that fungi also have resource and climate niches that are rooted in evolutionary history. Mean annual precipitation was positively correlated with relative abundance of fungi in both the Ascomycota and Zygomycota (Figure S3, Supporting Information), consistent with previous reports that these phyla correlate positively with precipitation and soil moisture (McHugh and Schwartz 2015; Zhao *et al*. 2017; Wang *et al*. 2018). Consistent with previous findings that different plant litter types host different fungal communities (Mincheva *et al*. 2014), litter type and tissue type were also significant correlates of relative abundance for Ascomycetes and Zygomycetes (Figure S4, Supporting Information). Initial C and N accounted for as much variation in Ascomycota and Zygomycota relative abundance as litter plant category (Figure S4 and Table S3, Supporting Information), suggesting that the correlation between relative abundance of these phyla and plant category is due to variation in growth resources for fungi among plant types (Osono and Takeda 2001). While initial C and N and litter tissue type did not correlate with the relative abundance of the Basidiomycota, it is possible that other aspects of resource availability for which we were not able to obtain data (e.g. soil pH and calcium content) could better explain the relative abundance of Basidiomycota, as they can correlate with fungal community composition in soil (Tedersoo *et al*. 2014; Purahong *et al*. 2016).

Consistent with our hypothesis that environmental variables would correlate more with the relative abundance of fungi at the genus level than at coarser taxonomic levels, plant category and tissue type correlated strongly with relative abundance of most fungal genera (Table S4, Supporting Information). These results are consistent with work showing differentiation in primary sources of C among fungal genera (Hibbett and Donoghue 2001; Hanson *et al*. 2008; McGuire *et al*. 2010; Tedersoo, May and Smith 2010; Nguyen *et al*. 2016), including brown rot fungi (Kerem, Jensen and Hammel 1999), plant pathogens (King *et al*. 2011), mycorrhizal fungi (Riley *et al*. 2014) and white rot fungi (Floudas *et al*. 2012). In line with this idea, we found that the relative abundance of white rot fungi was significantly correlated with plant category (Figure S5, Supporting Information). White rots were most abundant on coniferous litter, which could be due to the high initial lignin content of coniferous litter (>25%) (Melillo, Aber and Muratore 1982). While we did not find evidence that brown rot or white rot fungi varied predictably with decay stage (Figure S5, Supporting Information), it is possible that finer-scale differences in C-source preferences within or across these groups could account for genus-level patterns to succession.

Certain frequently observed genera (*Cladosporium, Flagellospora, Pestalotiopsis, Articulospora, Aureobasidium, Lemonniera*, *Mucor, Umbelopsis* and *Epicoccum*) showed significant decay-stage preferences that remained consistent across multiple biomes (Fig. 4; Table S4, Supporting Information). Some of these patterns parallel those reported in a large literature review of fungal succession (Kjøller and Struwe 2002); for example, we found that *Cladosporium* and *Epicoccum* are generally early colonizers, and that *Aureobasidium* tended to decline in relative abundance after mid decay. However, our data indicate that changes in these genera over time do not follow the hump-shaped trajectories that are traditionally referenced in discussions of succession (Fig. 1). In addition, we found that fungi in the genus *Mucor* prefer mid and late decay on average, whereas it has been reported in the literature as an early colonizer. Many of the other genera that have been anecdotally described as having distinct decay-stage preferences across studies (such as *Penicillium, Fusarium* and *Trichoderma*) (Kjøller and Struwe 2002) did not vary significantly across decay stages in our study. This discrepancy may be due to (i) bias in culture studies, which fail to pick up less easily cultured taxa, (ii) bias in DNA studies, in which inactive sequences are detected; or (iii) genera having different decay-stage preferences in different locations (Kjøller and Struwe 2002). Alternatively, early-colonizing fungi may shape subsequent community dynamics (i.e. priority effects) (Sridhar *et al*. 2009; Fukami *et al*. 2010; Peay, Belisle and Fukami 2012; Hiscox *et al*. 2015), either through competition (Hibbing *et al*. 2010; Lin *et al*. 2015) or facilitating the growth of other species (Datta *et al*. 2016). Traits related to competitive ability and resource use within the immediate community may therefore be some of the more rapidly evolving traits among fungal taxa that lead to succession dynamics within individual ecosystems.

## CONCLUSION

Laboratory and field studies show that different fungal species possess different abilities to break down plant biopolymers and have different effects on soil C cycling (Hanson *et al*. 2008; McGuire *et al*. 2010; Hättenschwiler, Fromin and Barantal 2011; Kubartová, Ottosson and Stenlid 2015; Treseder and Lennon 2015; Kyaschenko *et al*. 2017; Bhatnagar, Peay and Treseder 2018). Therefore, understanding the factors that control fungal community composition (Weber, Vilgalys and Kuske 2013) is critical to developing better predictions of decomposition rates and atmospheric C concentrations (Waldrop, Balser and Firestone 2000). We found a phylogenetic signal to succession of fungal decomposer communities across ecosystem types, such that different fungal phyla change in relative abundance throughout the decomposition process. These data indicate that, in addition to the critical role environmental factors play in fungal community assembly, evolutionary history plays a role in determining fungal succession regardless of ecosystem type or environmental condition. Incorporating fungal biology into predictive models of decomposition could therefore improve our simulations of terrestrial C cycle dynamics (Schmidt *et al*. 2011; Todd-Brown *et al*. 2012).

Because different fungal lineages have different trajectories during the decomposition process, we may be able to predict successional dynamics during decay based on the genetics of fungal decomposer species. Such predictions require further studies of the genomic differences among fungi that reach their highest relative abundance at early, middle and late decay, which could reveal the biological features of fungi that drive changes in abundances throughout decay. The results of this study also suggest that disturbance and environmental change that lead to the loss of whole lineages of fungi could also lead to potentially irreversible loss of some decomposer functions.

## Supplementary Material

fiz145_Supplemental_Files

## Notes

**Authorship:** JMB and SV designed the study; SV collected the data and carried out statistical and phylogenetic analyses; SV and JMB wrote the manuscript; and SV and JMB gave final approval for publication.

**Data accessibility:** All data are previously published in the meta-analysis source papers (Table S1).

## References

[bib1] Andrews JH, Kinkel LL, Berbee FM et al. Fungi, leaves, and the theory of island biogeography. Microb Ecol. 1987;3:277–90. 10.1007/BF02012947 24202721

[bib2] Aneja M, Sharma S, Fleischmann F et al. Microbial colonization of beech and spruce litter–influence of decomposition site and plant litter species on the diversity of microbial community. Microb Ecol. 2006;52:127–35. 16691328 10.1007/s00248-006-9006-3

[bib3] Bazzaz FA The physiological ecology of plant succession. Annu Rev Ecol Syst. 1979;10:351–71.

[bib4] Berg B, McClaugherty C Plant Litter: Decomposition, Humus Formation, Carbon Sequestration. Heidelberg: Springer, 2014.

[bib5] Bhatnagar JM, Peay KG, Treseder KK Litter chemistry influences decomposition through activity of specific microbial functional guilds. Ecol Monogr. 2018;88:429–44.

[bib6] Blackwell M, Vilgalys R, James TY et al. Eumycota: mushrooms, sac fungi, yeast, molds, rusts, smuts, etc. Tree of Life Web Project. 2012.

[bib7] Bollen WB Mulches and soil conditioners: carbon and nitrogen in farm and forest products. J Agric Food Chem. 1953;1:351–420.

[bib8] Bruder A, Salis RK, McHugh NJ et al. Multiple-stressor effects on leaf litter decomposition and fungal decomposers in agricultural streams contrast between litter species. Funct Ecol. 2016;30:1257–66.

[bib9] Carini P, Marsden PJ, Leff JW et al. Relic DNA is abundant in soil and obscures estimates of soil microbial diversity. Nat Microbiol. 2016;2:16242. 27991881 10.1038/nmicrobiol.2016.242

[bib10] Cornwell WK, Cornelissen JH, Amatangelo K et al. Plant species traits are the predominant control on litter decomposition rates within biomes worldwide. Ecol Lett. 2008;11:1065–71. 18627410 10.1111/j.1461-0248.2008.01219.x

[bib12] Crowther TW, Bradford MA Thermal acclimation in widespread heterotrophic soil microbes. Ecol Lett. 2013;16:469–77. 23331708 10.1111/ele.12069

[bib11] Crowther TW, Maynard DS, Thomas SM et al. Reply to Veresoglou: overdependence on “significance” testing in biology. Proc Natl Acad Sci USA. 2015;112:E5114. 26305960 10.1073/pnas.1513283112 PMC4577142

[bib13] Datta MS, Sliwerska E, Gore J et al. Microbial interactions lead to rapid micro-scale successions on model marine particles. Nat Commun. 2016;7:11965. 27311813 10.1038/ncomms11965 PMC4915023

[bib14] Enderlein G Miller, R. G.: simultaneous statistical inference. McGraw-Hill Book Comp., New York 1966, 272 S., 9 Tab., Preis 11.50. Biometr Z. 2007;12:363.

[bib15] Fernandez CW, Nguyen NH, Stefanski A et al. Ectomycorrhizal fungal response to warming is linked to poor host performance at the boreal-temperate ecotone. Glob Change Biol. 2017;23:1598–609. 10.1111/gcb.13510 27658686

[bib16] Fick SE, Hijmans RJ WorldClim 2: new 1-km spatial resolution climate surfaces for global land areas. Int J Climatol. 2017;37:4302–15.

[bib17] Fierer N, Lauber CL, Ramirez KS et al. Comparative metagenomic, phylogenetic and physiological analyses of soil microbial communities across nitrogen gradients. ISME J. 2012;6:1007–17. 22134642 10.1038/ismej.2011.159 PMC3329107

[bib18] Floudas D, Binder M, Riley R et al. The paleozoic origin of enzymatic lignin decomposition reconstructed from 31 fungal genomes. Science. 2012;336:1715–9. 22745431 10.1126/science.1221748

[bib19] Fogel R, Cromack K Jr. Effect of habitat and substrate quality on Douglas fir litter decomposition in western Oregon. Can J Bot. 1977;55:1632–40.

[bib21] Frankland JC Fungal succession—unravelling the unpredictable. Mycol Res. 1998;102:1–15.

[bib20] Frankland JC Succession of fungi on decaying petioles of Pteridium aquilinum. J Ecol. 1966;54:41–63.

[bib22] Fukami T, Dickie IA, Paula Wilkie J et al. Assembly history dictates ecosystem functioning: evidence from wood decomposer communities. Ecol Lett. 2010;13:675–84. 20412280 10.1111/j.1461-0248.2010.01465.x

[bib23] Gelman A, Hill J, Yajima M Why we (usually) don't have to worry about multiple comparisons. J Res Educ Eff. 2012;5:189–211.

[bib24] Giardina CP, Litton CM, Crow SE et al. Warming-related increases in soil CO2 efflux are explained by increased below-ground carbon flux. Nat Clim Change. 2014;4:822–7.

[bib25] Hanson CA, Allison SD, Bradford MA et al. Fungal taxa target different carbon sources in forest soil. Ecosystems. 2008;11:1157–67.

[bib26] Harmon LJ, Weir JT, Brock CD et al. GEIGER: investigating evolutionary radiations. Bioinformatics. 2008;24:129–31. 18006550 10.1093/bioinformatics/btm538

[bib27] Hättenschwiler S, Fromin N, Barantal S Functional diversity of terrestrial microbial decomposers and their substrates. C R Biol. 2011;334:393–402. 21640948 10.1016/j.crvi.2011.03.001

[bib28] Hawkes CV, Kivlin SN, Rocca JD et al. Fungal community responses to precipitation. Glob Change Biol. 2011;17:1637–45.

[bib30] Heitman J, Sun S, James TY Evolution of fungal sexual reproduction. Mycologia. 2013;105:1–27. 23099518 10.3852/12-253

[bib29] He W, Wu F, Yang W et al. Lignin degradation in foliar litter of two shrub species from the gap center to the closed canopy in an alpine fir forest. Ecosystems. 2016;19:115–28.

[bib31] Hibbett DS, Donoghue MJ Analysis of character correlations among wood decay mechanisms, mating systems, and substrate ranges in homobasidiomycetes. Syst Biol. 2001;50:215–42. 12116929

[bib32] Hibbett DS, Donoghue MJ, Olmstead R Analysis of character correlations among wood decay mechanisms, mating systems, and substrate ranges in homobasidiomycetes. Syst Biol. 2001;50:215–42. 12116929

[bib33] Hibbing ME, Fuqua C, Parsek MR et al. Bacterial competition: surviving and thriving in the microbial jungle. Nat Rev Microbiol. 2010;8:15–25. 19946288 10.1038/nrmicro2259 PMC2879262

[bib34] Hijmans RJ Raster: geographic data analysis and modeling. *R* package version 2.5-8 https://CRAN.R-project.org/package = raster, 2016, r948.

[bib35] Hiscox J, Savoury M, Müller CT et al. Priority effects during fungal community establishment in beech wood. ISME J. 2015;9:2246–60. 25798754 10.1038/ismej.2015.38 PMC4579477

[bib36] Hunt J, Boddy L, Randerson PF et al. An evaluation of 18S rDNA approaches for the study of fungal diversity in grassland soils. Microb Ecol. 2004;47:385–95. 14994180 10.1007/s00248-003-2018-3

[bib37] James FC, Rathbun S Rarefaction, relative abundance, and diversity of avian communities. Auk. 1981;98:785–800.

[bib38] James TY, Kauff F, Schoch CL et al. Reconstructing the early evolution of Fungi using a six-gene phylogeny. Nature. 2006;443:818–22. 17051209 10.1038/nature05110

[bib39] Kattge J, Díaz S, Lavorel S et al. TRY—a global database of plant traits. Glob Change Biol. 2011;17:2905–35.

[bib40] Kerem Z, Jensen KA, Hammel KE Biodegradative mechanism of the brown rot basidiomycete Gloeophyllum trabeum: Evidence for an extracellular hydroquinone-driven fenton reaction. FEBS Lett. 1999;446:49–54. 10100613 10.1016/s0014-5793(99)00180-5

[bib41] King BC, Waxman KD, Nenni NV et al. Arsenal of plant cell wall degrading enzymes reflects host preference among plant pathogenic fungi. Biotechnol Biofuels. 2011;4:4. 21324176 10.1186/1754-6834-4-4 PMC3051899

[bib42] Kjøller AH, Struwe S Fungal communities, succession, enzymes, and decomposition. Enzymes in the Environment—Activity, Ecology, and Applications. New York: Dekker 2002, 267–84.

[bib43] Kjøller R, Rosendahl S Cultivated and fallow fields harbor distinct communities of Basidiomycota. Fungal Ecol. 2014;9:43–51.

[bib44] Klotzbücher T, Kaiser K, Guggenberger G et al. A new conceptual model for the fate of lignin in decomposing plant litter. Ecology. 2011;92:1052–62. 21661566 10.1890/10-1307.1

[bib45] Koenig JE, Spor A, Scalfone N et al. Succession of microbial consortia in the developing infant gut microbiome. Proc Natl Acad Sci. 2011;108:4578–85. 20668239 10.1073/pnas.1000081107 PMC3063592

[bib46] Koren O, Knights D, Gonzalez A et al. A guide to enterotypes across the human body: meta-analysis of microbial community structures in human microbiome datasets. PLoS Comput Biol. 2013;9:e1002863. 23326225 10.1371/journal.pcbi.1002863 PMC3542080

[bib47] Kubartová A, Ottosson E, Stenlid J Linking fungal communities to wood density loss after 12 years of log decay. FEMS Microbiol Ecol. 2015;91:fiv032. 25873458 10.1093/femsec/fiv032

[bib48] Kurtzman CP, Fell JW, Boekhout T The Yeasts: A Taxonomic Study, 5th edn, Burlington: Elsevier Science, 2011.

[bib49] Kyaschenko J, Clemmensen KE, Hagenbo A et al. Shift in fungal communities and associated enzyme activities along an age gradient of managed Pinus sylvestris stands. ISME J. 2017;11:863–74. 28085155 10.1038/ismej.2016.184 PMC5364365

[bib50] Lal R, Follett RF Terrestrial carbon sequestration potential in reclaimed mine land ecosystems to mitigate the greenhouse effect. Waste Manage. 2009;250:164.165–134.164.

[bib52] Lindner DL, Vasaitis R, Kubartová A et al. Initial fungal colonizer affects mass loss and fungal community development in Picea abies logs 6yr after inoculation. Fungal Ecol. 2011;4:449–60.

[bib51] Lin Y, He X, Ma T et al. Priority colonization of Cinnamomum camphora litter by endophytes affects decomposition rate, fungal community and microbial activities under field conditions. Pedobiologia. 2015;58:177–85.

[bib53] Looby CI, Treseder KK Shifts in soil fungi and extracellular enzyme activity with simulated climate change in a tropical montane cloud forest. Soil Biol Biochem. 2018;117:87–96.

[bib54] Losos JB Phylogenetic niche conservatism, phylogenetic signal and the relationship between phylogenetic relatedness and ecological similarity among species. Ecol Lett. 2008;11:995–1003. 18673385 10.1111/j.1461-0248.2008.01229.x

[bib55] MacArthur RH, Wilson EO The theory of island biogeography. Monographs in Population Biology. Princeton University Press 1967.

[bib56] Manzoni S, Porporato A Soil carbon and nitrogen mineralization: theory and models across scales. Soil Biol Biochem. 2009;41:1355–79.

[bib57] Marín-Martínez F, Sánchez-Meca J Weighting by inverse variance or by sample size in random-effects meta-analysis. Educ Psychol Meas. 2010;70:56–73.

[bib58] McGuire KL, Bent E, Borneman J et al. Functional diversity in resource use by fungi. Ecology. 2010;91:2324–32. 20836454 10.1890/09-0654.1

[bib59] McHugh TA, Schwartz E Changes in plant community composition and reduced precipitation have limited effects on the structure of soil bacterial and fungal communities present in a semiarid grassland. Plant Soil. 2014;388:175–86.

[bib60] Meier CL, Bowman WD Chemical composition and diversity influence non-additive effects of litter mixtures on soil carbon and nitrogen cycling: Implications for plant species loss. Soil Biol Biochem. 2010;42:1447–54.

[bib61] Melillo JM, Aber JD, Muratore JF Nitrogen and lignin control of hardwood leaf litter decomposition dynamics. Ecology. 1982;63:621–6.

[bib62] Mincheva T, Barni E, Varese GC et al. Litter quality, decomposition rates and saprotrophic mycoflora in Fallopia japonica (Houtt.) Ronse Decraene and in adjacent native grassland vegetation. Acta Oecol. 2014;54:29–35.

[bib63] Mönkkönen M, Reunanen P, Kotiaho JS et al. Cost-effective strategies to conserve boreal forest biodiversity and long-term landscape-level maintenance of habitats. Eur J For Res. 2011;130:717–27.

[bib64] Morgenstern I, Klopman S, Hibbett DS Molecular evolution and diversity of lignin degrading heme peroxidases in the agaricomycetes. J Mol Evol. 2008;66:243–57. 18292958 10.1007/s00239-008-9079-3

[bib65] Mundra S, Halvorsen R, Kauserud H et al. Ectomycorrhizal and saprotrophic fungi respond differently to long-term experimentally increased snow depth in the High Arctic. MicrobiologyOpen. 2016;5:856–69. 27255701 10.1002/mbo3.375 PMC5061721

[bib66] Nguyen NH, Song Z, Bates ST et al. FUNGuild: an open annotation tool for parsing fungal community datasets by ecological guild. Fungal Ecol. 2016;20:241–8.

[bib67] Norros V, Halme P Growth sites of polypores from quantitative expert evaluation: Late-stage decayers and saprotrophs fruit closer to ground. Fungal Ecol. 2017;28:53–65.

[bib68] Odum EP The strategy of ecosystem development. Science. 1969;164:262–70. 5776636 10.1126/science.164.3877.262

[bib69] Osono T Ecology of ligninolytic fungi associated with leaf litter decomposition. Ecol Res. 2007;22:955–74.

[bib70] Osono T, Ishii Y, Takeda H et al. Fungal succession and lignin decomposition on Shorea obutsa leaves in a tropical seasonal forest in northern Thailand. Fungal Divers. 2009;36:101–19.

[bib71] Osono T, Takeda H Organic chemical and nutrient dynamics in decomposing beech leaf litter in relation to fungal ingrowth and succession during 3-year decomposition processes in a cool temperate deciduous forest in Japan. Ecol Res. 2001;16:649–70.

[bib72] Ottosson E, Nordén J, Dahlberg A et al. Species associations during the succession of wood-inhabiting fungal communities. Fungal Ecol. 2014;11:17–28.

[bib73] Pasqualetti M, Mulas B, Zucconic L et al. Succession of microfungal communities on Myrtus communis leaf litter in a Sardinian Mediterranean maquis ecosystem. Mycol Res. 1999;103:724–8.

[bib74] Peay KG, Belisle M, Fukami T Phylogenetic relatedness predicts priority effects in nectar yeast communities. Proc Biol Sci. 2012;279:749–58. 21775330 10.1098/rspb.2011.1230 PMC3248732

[bib75] Peet RK The measurement of species diversity. Annu Rev Ecol Syst. 1974;5:285–307.

[bib76] Promputtha I, Lumyong S, Dhanasekaran V et al. A phylogenetic evaluation of whether endophytes become saprotrophs at host senescence. Microb Ecol. 2007;53:579–90. 17410394 10.1007/s00248-006-9117-x

[bib77] Purahong W, Wubet T, Lentendu G et al. Life in leaf litter: novel insights into community dynamics of bacteria and fungi during litter decomposition. Mol Ecol. 2016;25:4059–74. 27357176 10.1111/mec.13739

[bib78] Purschke O, Schmid BC, Sykes MT et al. Contrasting changes in taxonomic, phylogenetic and functional diversity during a long-term succession: Insights into assembly processes. J Ecol. 2013;101:857–66.

[bib79] Quast C, Pruesse E, Yilmaz P et al. The SILVA ribosomal RNA gene database project: improved data processing and web-based tools. Nucleic Acids Res. 2013;41:D590–6. 23193283 10.1093/nar/gks1219 PMC3531112

[bib81] Rayner ADM, Boddy L Fungal Decomposition of Wood. Its Biology and Ecology. Chichester, Sussex: John Wiley & Sons, 1988.

[bib80] R Development Core Team. R: a language and environment for statistical computing. R Foundation for Statistical Computing. 2011 doi:10.1007/978-3-540-74686-7.

[bib82] Riley R, Salamov AA, Brown DW et al. Extensive sampling of basidiomycete genomes demonstrates inadequacy of the white-rot/brown-rot paradigm for wood decay fungi. Proc Natl Acad Sci USA. 2014;111:9923–8. 24958869 10.1073/pnas.1400592111 PMC4103376

[bib83] Rizzo AM, Pang K-L New primers for detection of Smittium spp. (Trichomycetes, Zygomycota) in insect hosts. Fungal Divers. 2005;19:129–36.

[bib84] Rohatgi A WebPlotDigitizer User Manual Version 3.4, http://arohatgi. info/WebPlotDigitizer/app, 2014; 1–18.. Available at: http://arohatgi.info/WebPlotDigitizer/userManual.pdf.

[bib85] Rothman KJ No adjustments are needed for multiple comparisons. Epidemiology. 1990;1:43–6. 2081237

[bib86] Sayer EJ, Oliver AE, Fridley JD et al. Links between soil microbial communities and plant traits in a species-rich grassland under long-term climate change. Ecol Evol. 2017;7:855–62. 28168022 10.1002/ece3.2700 PMC5288249

[bib87] Schmidt MW, Torn MS, Abiven S et al. Persistence of soil organic matter as an ecosystem property. Nature. 2011;478:49–56. 21979045 10.1038/nature10386

[bib88] Sinsabaugh RL, Belnap J, Findlay SG et al. Extracellular enzyme kinetics scale with resource availability. Biogeochemistry. 2014;121:287–304.

[bib89] Smith ME, Douhan GW, Rizzo DM Intra-specific and intra-sporocarp ITS variation of ectomycorrhizal fungi as assessed by rDNA sequencing of sporocarps and pooled ectomycorrhizal roots from a *Quercus* woodland. Mycorrhiza. 2007;18:15–22. 17710446 10.1007/s00572-007-0148-z

[bib90] Spatafora JW, Chang Y, Benny GL et al. A phylum-level phylogenetic classification of zygomycete fungi based on genome-scale data. Mycologia. 2016;108:1028–46. 27738200 10.3852/16-042 PMC6078412

[bib91] Sridhar KR, Duarte S, Cássio F et al. The role of early fungal colonizers in leaf-litter decomposition in portuguese streams impacted by agricultural runoff. Int Rev Hydrobiol. 2009;94:399–409.

[bib92] Stamatakis A RAxML version 8: a tool for phylogenetic analysis and post-analysis of large phylogenies. Bioinformatics. 2014;30:1312–3. 24451623 10.1093/bioinformatics/btu033 PMC3998144

[bib93] Swenson NG, Enquist BJ Opposing assembly mechanisms in a Neotropical dry forest: Implications for phylogenetic and functional community ecology. Ecology. 2009;90:2161–70. 19739378 10.1890/08-1025.1

[bib95] Talbot JM Fungal communities and climate change. In: Dighton J, White JG(eds). The Fungal Community: Its Organization and Role in the Ecosystem. 4th edn Boca Raton, FL: Taylor & Francis Group, LLC, 2017.

[bib94] Talbot JM, Bruns TD, Taylor JW et al. Endemism and functional convergence across the North American soil mycobiome. Proc Natl Acad Sci USA. 2014;111:6341–6. 24733885 10.1073/pnas.1402584111 PMC4035912

[bib96] Tanabe Y, O'Donnell K, Saikawa M et al. Molecular phylogeny of parasitic Zygomycota (Dimargaritales, Zoopagales) based on nuclear small subunit ribosomal DNA sequences. Mol Phylogenet Evol. 2000;16:253–62. 10942611 10.1006/mpev.2000.0775

[bib97] Tedersoo L, Bahram M, Põlme S et al. Global diversity and geography of soil fungi. Science. 2014;346:1256688. 25430773 10.1126/science.1256688

[bib98] Tedersoo L, May TW, Smith ME Ectomycorrhizal lifestyle in fungi: global diversity, distribution, and evolution of phylogenetic lineages. Mycorrhiza. 2010;20:217–63. 20191371 10.1007/s00572-009-0274-x

[bib99] Todd-Brown KEO, Hopkins FM, Kivlin SN et al. A framework for representing microbial decomposition in coupled climate models. Biogeochemistry. 2012;109:19–33.

[bib100] Treseder KK, Bent E, Borneman J et al. Shifts in fungal communities during decomposition of boreal forest litter. Fungal Ecol. 2014;10:58–69.

[bib101] Treseder KK, Lennon JT Fungal traits that drive ecosystem dynamics on land. Microbiol Mol Biol Rev. 2015;79:243–62. 25971588 10.1128/MMBR.00001-15 PMC4429240

[bib102] Urbanová M, Šnajdr J, Baldrian P Composition of fungal and bacterial communities in forest litter and soil is largely determined by dominant trees. Soil Biol Biochem. 2015;84:53–64.

[bib103] Venables WN, Ripley BD Modern Applied Statistics with S. 4th edn New York: Springer, 2002.

[bib104] Vevea JL, Hedges LV A general linear model for estimating effect size in the presence of publication bias. Psychometrika. 1995;60:419–35.

[bib105] Voříšková J, Baldrian P Fungal community on decomposing leaf litter undergoes rapid successional changes. ISME J. 2013;7:477–86. 23051693 10.1038/ismej.2012.116 PMC3578564

[bib106] Waldrop MP, Balser TC, Firestone MK Linking microbial community composition to function in a tropical soil. Soil Biol Biochem. 2000;32:1837–46.

[bib107] Wang D, Rui Y, Ding K et al. Precipitation drives the biogeographic distribution of soil fungal community in Inner Mongolian temperate grasslands. J Soils Sediments. 2018;18:222–8.

[bib108] Warton DI, Guillaume Blanchet F, O'Hara RB et al. So many variables: joint modeling in community ecology. Trends Ecol Evol. 2015;30:766–79. 26519235 10.1016/j.tree.2015.09.007

[bib109] Webb CO, Ackerly DD, Kembel SW Phylocom: software for the analysis of phylogenetic community structure and trait evolution. Bioinformatics. 2008;24:2098–100. 18678590 10.1093/bioinformatics/btn358

[bib110] Weber CF, Vilgalys R, Kuske CR Changes in fungal community composition in response to elevated atmospheric CO2 and nitrogen fertilization varies with soil horizon. Front Microbiol. 2013;4:78. 23641237 10.3389/fmicb.2013.00078 PMC3621283

[bib111] Webster J, Weber R Introduction to Fungi. Cambridge University Press, 2007.

[bib112] White MM, James TY, O'Donnell K et al. Phylogeny of the Zygomycota based on nuclear ribosomal sequence data. Mycologia. 2006;98:872–84. 17486964 10.3852/mycologia.98.6.872

[bib113] Wilson KB, Baldocchi DD, Hanson PJ Spatial and seasonal variability of photosynthetic parameters and their relationship to leaf nitrogen in a deciduous forest. Tree Physiol. 2000;20:565–78. 12651421 10.1093/treephys/20.9.565

[bib114] Wood S Generalized additive models: an introduction with R. J Am Statist Assoc. 2007;102:760–1.

[bib115] Wright JP, Sutton-Grier A Does the leaf economic spectrum hold within local species pools across varying environmental conditions?. Funct Ecol. 2012;26:1390–8.

[bib116] Yan X, Su XG Linear Regression Analysis. Hackensack, NJ: World Scientific, 2009.

[bib117] Yee TW, Mitchell ND Generalized additive models in plant ecology. J Veg Sci. 1991;2:587–602.

[bib118] Yguel B, Bailey R, Tosh ND et al. Phytophagy on phylogenetically isolated trees: Why hosts should escape their relatives. Ecol Lett. 2011;14:1117–24. 21923895 10.1111/j.1461-0248.2011.01680.x

[bib119] Zhang D, Hui D, Luo Y et al. Rates of litter decomposition in terrestrial ecosystems: global patterns and controlling factors. J Plant Ecol. 2008;1:85–93.

[bib120] Zhao Q, Jian S, Nunan N et al. Altered precipitation seasonality impacts the dominant fungal but rare bacterial taxa in subtropical forest soils. Biol Fertil Soils. 2017;53:231–45.

