# Supplementary material for: An evolutionary signal to fungal succession during plant litter decay
Source: FEMS Microbiol Ecol. 2019 Sep 7;95(10):fiz145. doi: 10.1093/femsec/fiz145 (PMC6772037; doi:10.1093/femsec/fiz145)
Supplement: fiz145_Supplemental_Files [file fiz145_supplemental_files.zip › SupplementaryFigures_August_2019.docx]

Fig. S1. Phylogenetic tree of fungi constructed at the genus level using 28S rDNA. Branches are color coded according to the percent mass loss at which each genus reaches its highest weighted average relative abundance.

Fig. S2. Relative contribution of each fungal phylum to the total relative abundance of all fungi at each decay stage detected across studies. Early decay was defined as <25% mass loss, middle decay was defined as 25-35% mass loss, and late decay was defined as >35% mass loss.

Fig. S3. A-B. Relative abundance of Ascomycota, Basidiomycota, and Zygomycota plotted against A. mean annual temperature in degrees Celsius or B. mean annual precipitation in millimeters. Statistics represent the results of generalized linear single regression models.

Fig. S4. A-D. Relative abundance of Ascomycota, Basidiomycota, and Zygomycota plotted against A. litter plant category, B. litter tissue type, C. initial litter percent C, or D. initial litter percent N. Statistics represent the results of generalized linear single regression models.

A. B. C.

Fig. S5. Relationship between percent mass loss (A) or litter plant type and relative abundance of white rot (B) and brown rot (C) fungi. Statistics for linear regression (A) are derived from multiple regression generalized linear models. Statistics for plant type analyses (B, C) are derived from one-way analysis of variance. Letters denote Tukey groupings across plant categories at p<0.05.

Fig. S6. Weighted relative abundance of fungal endophytes and non-endophytes plotted against percent litter mass loss. Lines represent linear model trend lines. Each point represents an individual sampling time point on a litter type in a published study (n=10-28). P-values and adjusted R^2^ values represent the result of single-regression generalized additive models.
