# Supplementary material for: An evolutionary signal to fungal succession during plant litter decay
Source: FEMS Microbiol Ecol. 2019 Sep 7;95(10):fiz145. doi: 10.1093/femsec/fiz145 (PMC6772037; doi:10.1093/femsec/fiz145)
Supplement: fiz145_Supplemental_Files [file fiz145_supplemental_files.zip › TablesS2andS3.docx]

| Table S2. Multiple regression model final model statistics for Ascomycota, Basidiomycota, and Zygomycota. Final models were obtained using stepAIC in R’s MASS package (Venables and Ripley, 2002). |
| --- |

| Ascomycota Multiple Regression Final Model, All Data | |  |  |
| --- | --- | --- | --- |
| Variable Name | Variable Description | Model R2 | Model P Value |
| *Percent mass loss* | Percent original litter mass lost | 0.56 | **<0.001** |
| *MAT* | Mean annual temperature |  |  |
| *MAP* | Mean annual precipitation |  |  |
| *Tissue* | Litter tissue type (leaf/stem) |  |  |
| *Plant category* | Deciduous/coniferous/grass/shrub |  |  |
| *Data type* | Sequence vs. culture study |  |  |
|  | |  |  |
| Ascomycota Multiple Regression Final Model, DNA Sequence Data | |  |  |
| Variable Name | Variable Description | Model R2 | Model P Value |
| *Percent mass loss* | Percent original litter mass lost | 0.68 | **<0.001** |
| *MAT* | Mean annual temperature |  |  |
| *MAP* | Mean annual precipitation |  |  |
| *Tissue* | Litter tissue type (leaf/stem) |  |  |
| *Plant category* | Deciduous/coniferous/grass/shrub |  |  |
|  | |  |  |
| Ascomycota Multiple Regression Final Model, Culture Data | |  |  |
| Variable Name | Variable Description | Model R2 | Model P Value |
| *Percent mass loss* | Percent original litter mass lost | 0.34 | **<0.001** |
| *MAT* | Mean annual temperature |  |  |
| *MAP* | Mean annual precipitation |  |  |
| *Tissue* | Litter tissue type (leaf/stem) |  |  |
| *Plant category* | Deciduous/coniferous/grass/shrub |  |  |
|  |  |  |  |
|  | |  |  |
| Basidiomycota Multiple Regression Final Model, Sequence Data | |  |  |
| Variable Name | Variable Description | Model R2 | Model P Value |
| *Plant category* | Deciduous/coniferous/grass/shrub | 0.10 | 0.10 |
|  |  |  |  |
| Zygomycota Multiple Regression Final Model, Sequence Data | |  |  |
| Variable Name | Variable Description | Model R2 | Model P Value |
| *Percent mass loss* | Percent original litter mass lost | 0.61 | **<0.001** |
| *MAP* | Mean annual precipitation |  |  |
| *Tissue* | Litter tissue type (leaf/stem) |  |  |
| *Plant category* | Deciduous/coniferous/grass/shrub |  |  |
|  |  |  |  |

Table S3. Final generalized linear multiple regression model statistics for Ascomycota, Basidiomycota, and Zygomycota with initial percent litter N and initial percent litter C substituted for litter plant category and litter tissue type.

| Ascomycota Multiple Regression Final Model, Sequence Data | |  |  |
| --- | --- | --- | --- |
| Variable Name | Variable Description | Model R2 | Model P Value |
| *Percent mass loss* | Percent original litter mass lost | 0.93 | **<0.001** |
| *MAT* | Mean annual temperature |  |  |
| *MAP* | Mean annual precipitation |  |  |
| *Initial C* | Initial litter percent C |  |  |
| *Initial N* | Initial litter percent N |  |  |
| *Data type* | Sequence vs. culture study |  |  |
|  |  |  |  |
|  |  |  |  |
|  | |  |  |
| Basidiomycota Multiple Regression Final Model | |  |  |
| Variable Name | Variable Description | Model R2 | Model P Value |
| *Initial C* | Initial litter percent C | 0.027 | 0.53 |
| *Initial N* | Initial litter percent N |  |  |
|  |  |  |  |
|  |  |  |  |
|  | |  |  |
| Zygomycota Multiple Regression Final Model | |  |  |
| Variable Name | Variable Description | Model R2 | Model P Value |
| *Percent mass loss* | Percent original litter mass lost | 0.52 | **<0.001** |
| *MAP* | Mean annual precipitation |  |  |
| *Initial C* | Initial litter percent C |  |  |
| *Initial N* | Initial litter percent N |  |  |
|  |  |  |  |
