# Supplementary figures and images for: An evolutionary signal to fungal succession during plant litter decay
Source: FEMS Microbiol Ecol. 2019 Sep 7;95(10):fiz145. doi: 10.1093/femsec/fiz145 (PMC6772037; doi:10.1093/femsec/fiz145)

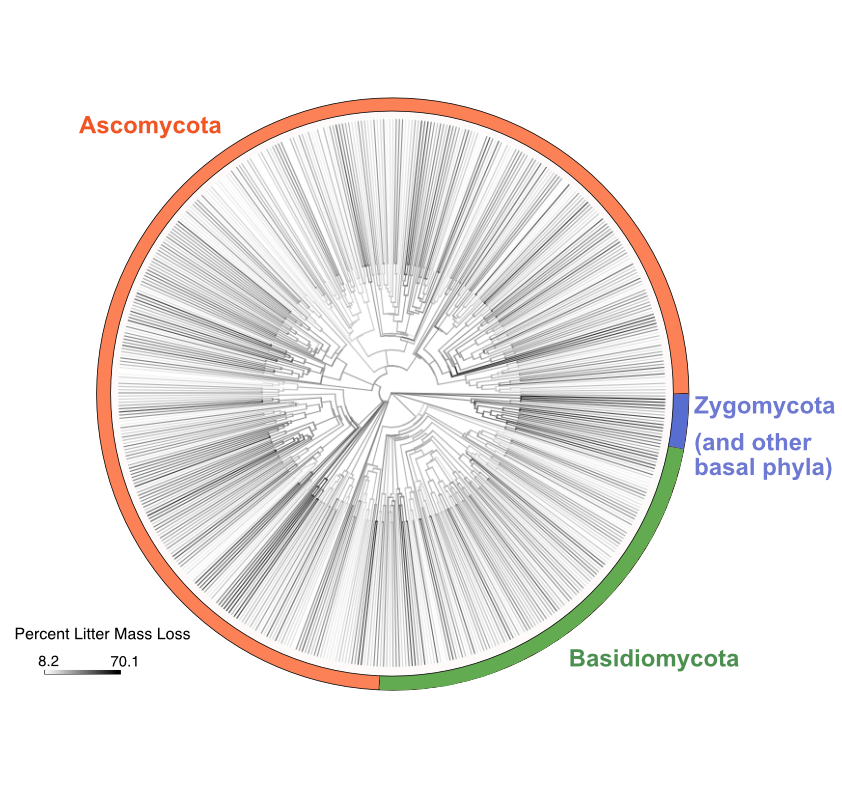

Supplement: fiz145_Supplemental_Files [file fiz145_supplemental_files.zip › Figure_S1_Sept_2019_ViveloBhatnagar.tiff]

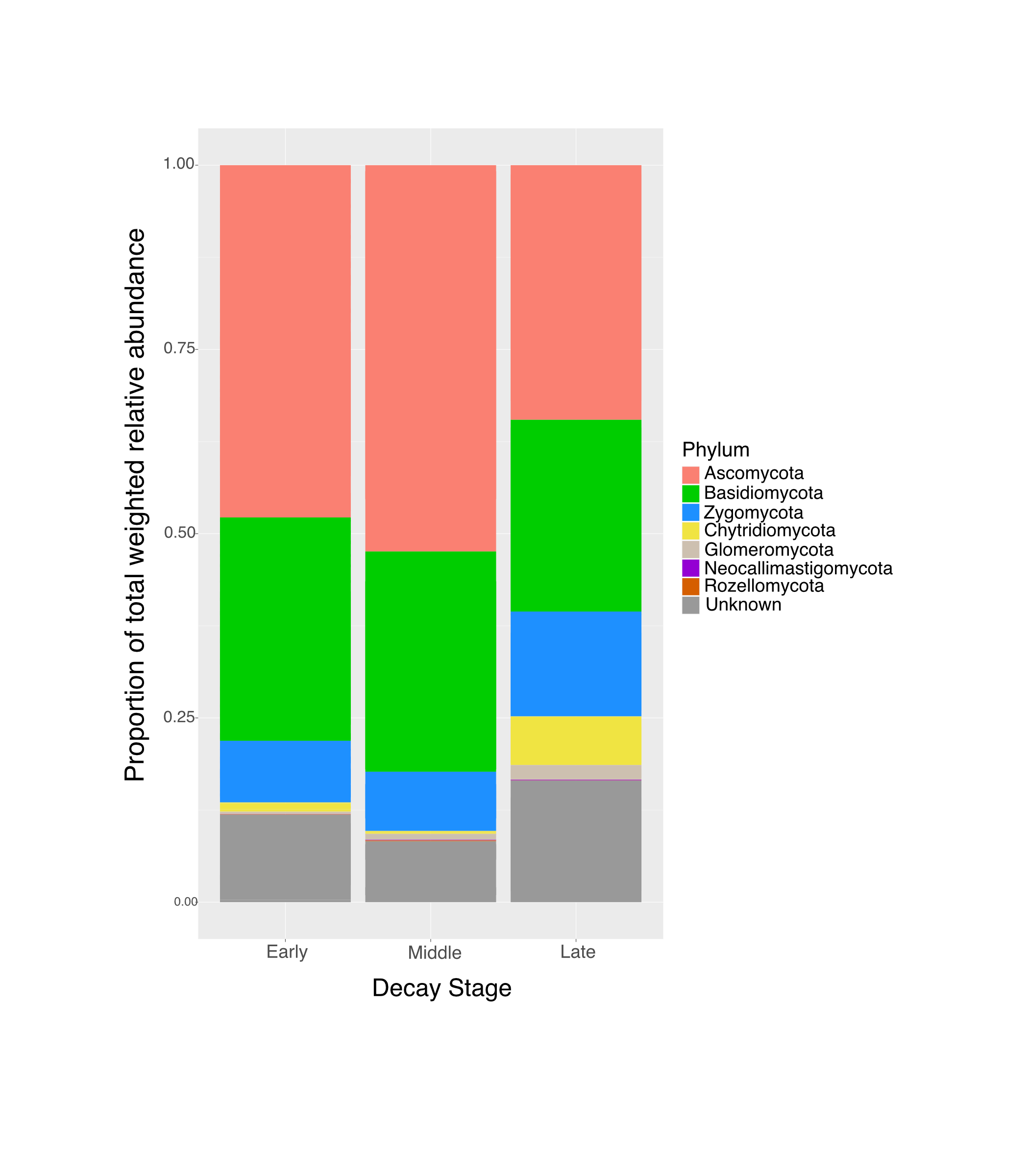

Supplement: fiz145_Supplemental_Files [file fiz145_supplemental_files.zip › Figure_S2_Sept_2019_ViveloBhatnagar.tiff]

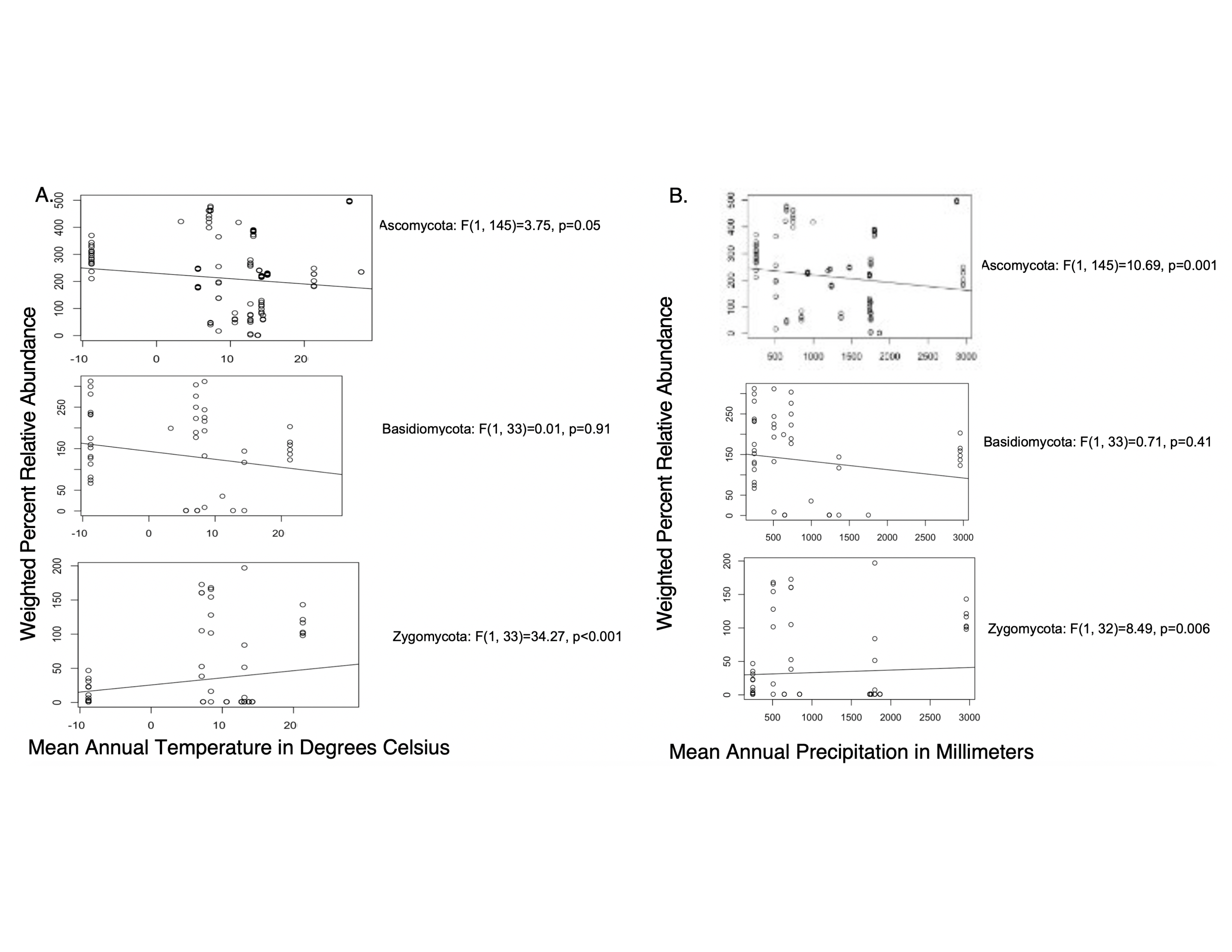

Supplement: fiz145_Supplemental_Files [file fiz145_supplemental_files.zip › Figure_S3_Sept_2019_ViveloBhatnagar.tiff]

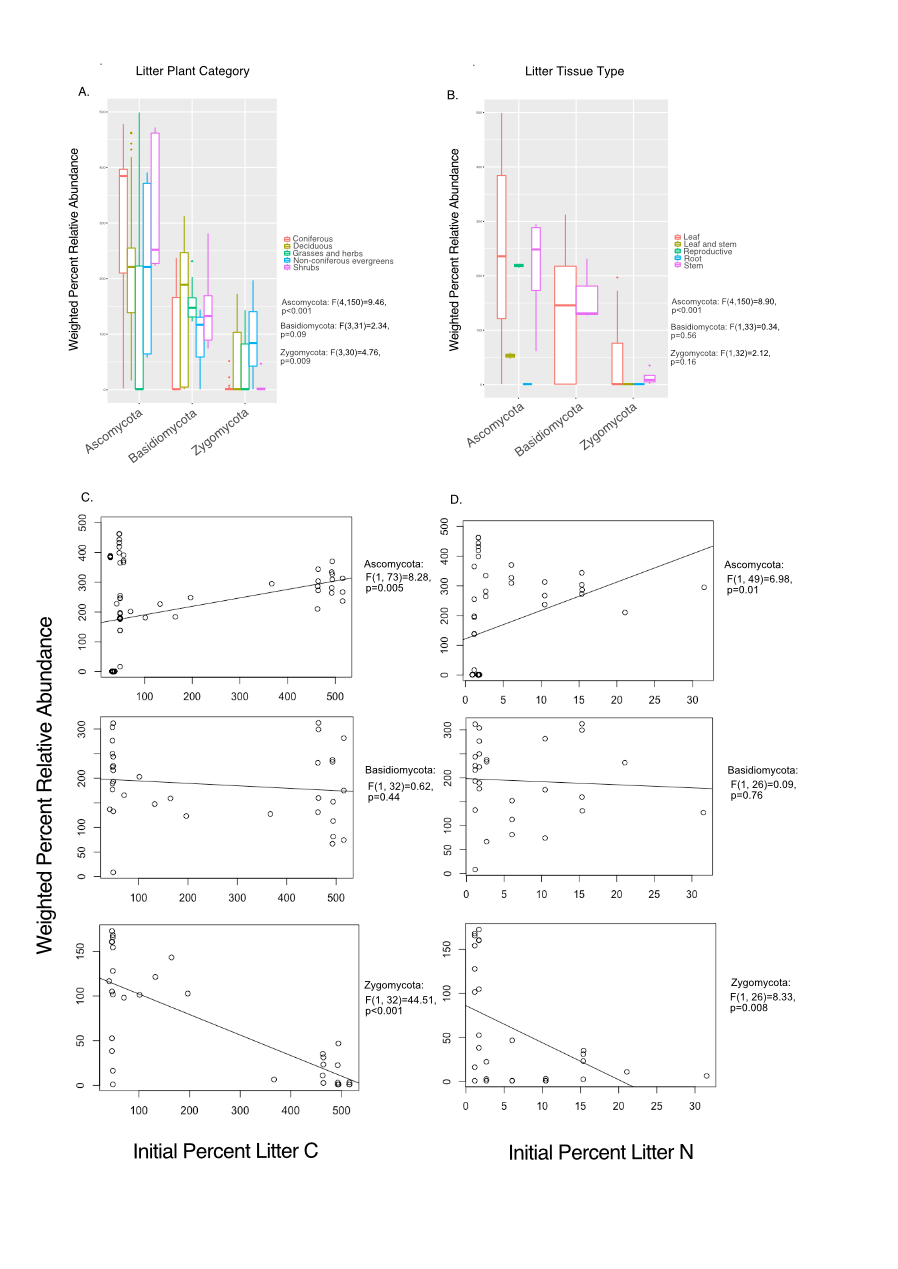

Supplement: fiz145_Supplemental_Files [file fiz145_supplemental_files.zip › Figure_S4_Sept_2019_ViveloBhatnagar.tiff]

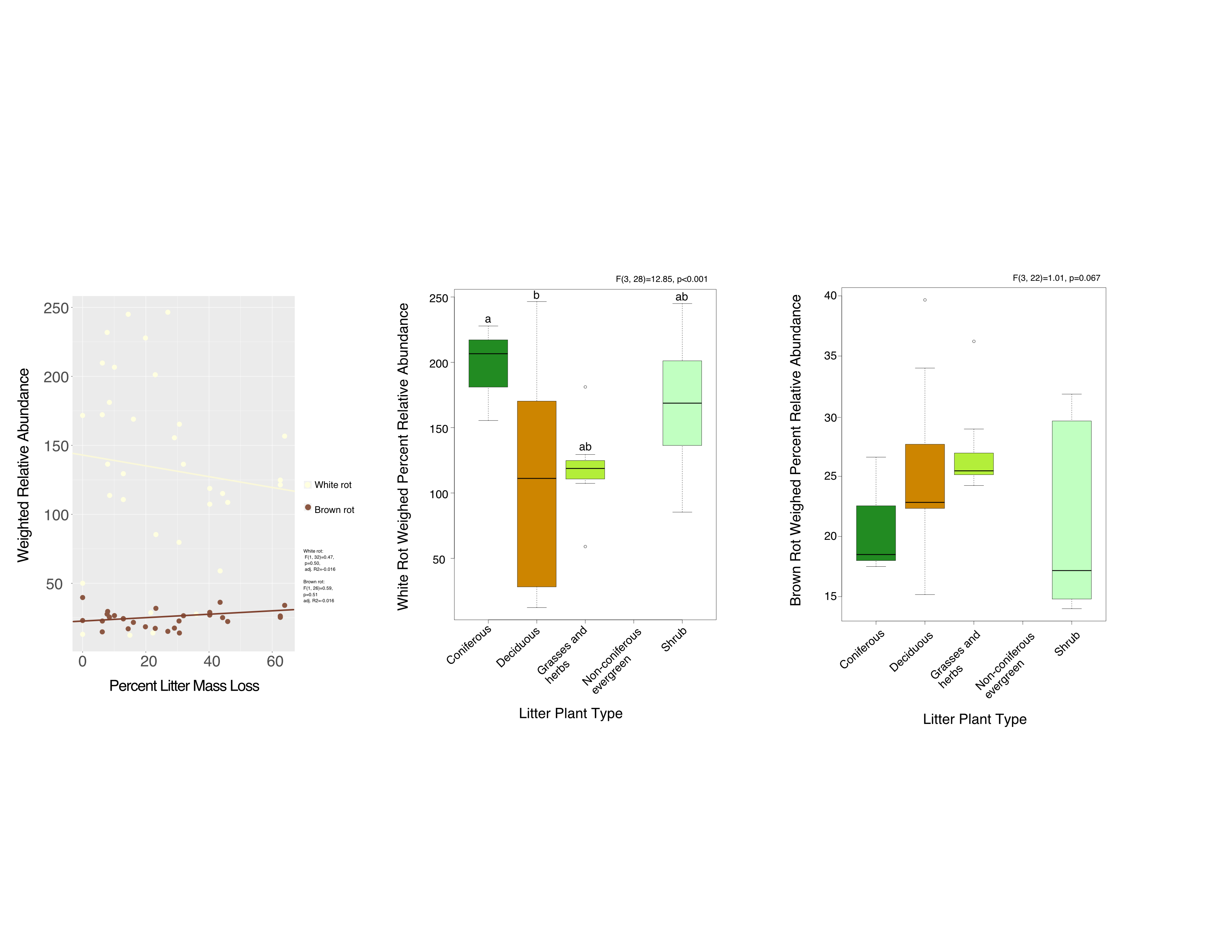

Supplement: fiz145_Supplemental_Files [file fiz145_supplemental_files.zip › Figure_S5_Sept_2019_ViveloBhatnagar.tiff]

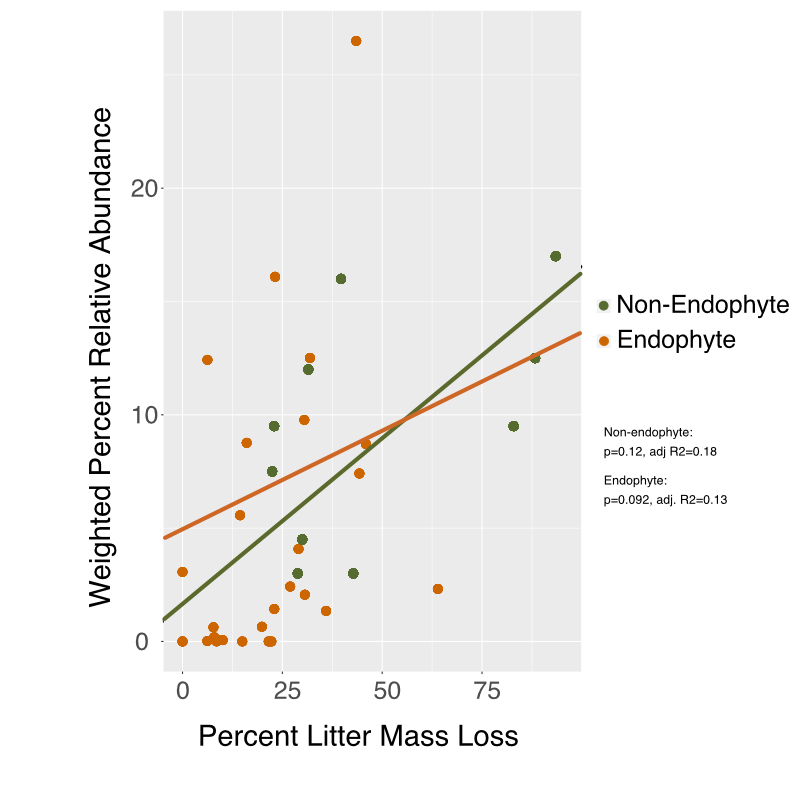

Supplement: fiz145_Supplemental_Files [file fiz145_supplemental_files.zip › Figure_S6_Sept_2019_ViveloBhatnagar.tiff]
